# Single chamber air–cathode microbial fuel cells as biosensors for determination of biodegradable organics

**DOI:** 10.1007/s10529-019-02668-4

**Published:** 2019-04-03

**Authors:** Bálint Lóránt, Miklós Gyalai-Korpos, Igor Goryanin, Gábor Márk Tardy

**Affiliations:** 1 Department of Applied Biotechnology and Food Science, Budapest University of Technology and Economics, Szt. Gellért tér 4, Budapest, 1111 Hungary; 2 Pannon Pro Innovations Ltd, P.O.B 41, Budapest, 1400 Hungary; 3 BES Europe Ltd, Murányi u. 39, Budapest, 1078 Hungary; 4 School of Informatics, University of Edinburgh, 10 Crichton str, Edinburgh, EH8 9AB UK; 5 Okinawa Institute of Science and Technology Graduate University, 1919-1 Tancha, Onna-Son, Kunigami-gun, Okinawa, 904-0495 Japan; 6 Tianjin Institute of Industrial Biotechnology, 32 West 7th Avenue, Tianjin Airport Economic Area, Tianjin, 300308 China

**Keywords:** Air cathode, Biosensor, Biodegradation kinetics, Microbial fuel cell

## Abstract

**Objectives:**

Single chamber air cathode microbial fuel cells (MFCs) were investigated with sodium-acetate and peptone as test substrates to assess the potential for application as biosensor to determine the concentration of biodegradable organics in water/wastewater samples.

**Results:**

MFCs provided well-reproducible performance at high (> 2000 mg COD l^−1^—Chemical Oxygen Demand) acetate concentration values. Current in the cells proved to be steady from 25 to 35 °C, significant decrease was, however, revealed in the current below 20 °C. Direct calculation of non-toxic biodegradable substrate concentration in water/wastewater from the current in MFCs is possible only in the non-saturated substrate concentration range due to the Monod-like dependence of the current. This range was determined by a fitted and verified Monod-based kinetic model. Half saturation constant (K_S_) values were calculated at 30 °C applying different external resistance values (100 Ω, 600 Ω and 1000 Ω, respectively). In each case K_S_ remained below 10 mg COD l^−1^.

**Conclusions:**

Biosensors with this particular MFC design and operation are potentially applicable for detecting as low as 5 mg COD l^−1^ readily biodegradable substrates, and measuring the concentration of these substances up to ~ 50–70 mg COD l^−1^.

## Introduction

Microbial fuel cells (MFCs) are special bioreactors that can convert the chemical energy stored in biodegradable substances directly into electricity. The operating principle of an MFC is based on the metabolism of exoelectrogenic bacteria: these organisms can transfer the electrons gained through biodegradation of organic compounds to the solid anode, thus generating electricity in the external circuit of the cell. MFCs have multiple fields of potential application: they can be implemented as energy-efficient wastewater treatment technologies or used as power source for portable devices with low energy demand (Fedorovich et al. 2009; Rahimnejad et al. 2015; Divyalakshmi et al. 2017). Yet, one of the most promising way of utilizing MFCs is the application as biosensors to detect biodegradable organics and/or toxic compounds even at extremely low concentrations (Abrevaya et al. 2015a, b; Sun et al. 2015).

The basic way of measuring the concentration of non-toxic biodegradable organic matter in water or wastewater with MFCs is based on the correlation between the generated current and substrate concentration in the anolyte, which can be described generally with Monod-like kinetics (Lóránt et al. 2015), as shown in Eq. 1.

1 $$I = I_{max} \frac{S}{{K_{s} + S}}$$

where I is the measured current in the circuit of the cell (µA), I_max_ is the maximum current produced at high substrate concentrations (µA), S is the bidegradable substrate concentration (mg COD l^−1^) and K_S_ is the half-saturation constant (mg COD l^−1^).

Because of the saturation curve, direct determination of the substrate concentration in the anolyte by measuring current is possible only in the non-saturated substrate concentration range, therefore it is essential to know the value of the main parameter affecting the shape of the Monod curve: the half saturation constant (K_S_). Based on the literature, K_S_ varies in a wide range (from < 1 to > 100 mg COD l^−1^), depending on the design and operation (Tront et al. 2008; Sharma and Li 2010; Tardy et al. 2017a). As a result, it is possible to develop MFC-based biosensors for working in trace amount of substrate concentration range (0–150 µM of acetate) (Quek et al. 2015), to higher organic matter content (3 to 164 mg COD l^−1^) (Di Lorenzo et al. 2014).

Single chamber air cathode MFCs were assembled and investigated in this research applying noble metal free biomass originated air cathode materials to determine the potential of this design as a biosensor. Hydraulic and biokinetic model of the cells were developed, fitted and verified for the appropriate interpretation of the cell’s electric response to substrate concentration changes.

## Materials and methods

### MFC architecture, data collection

**Fig. 1:**
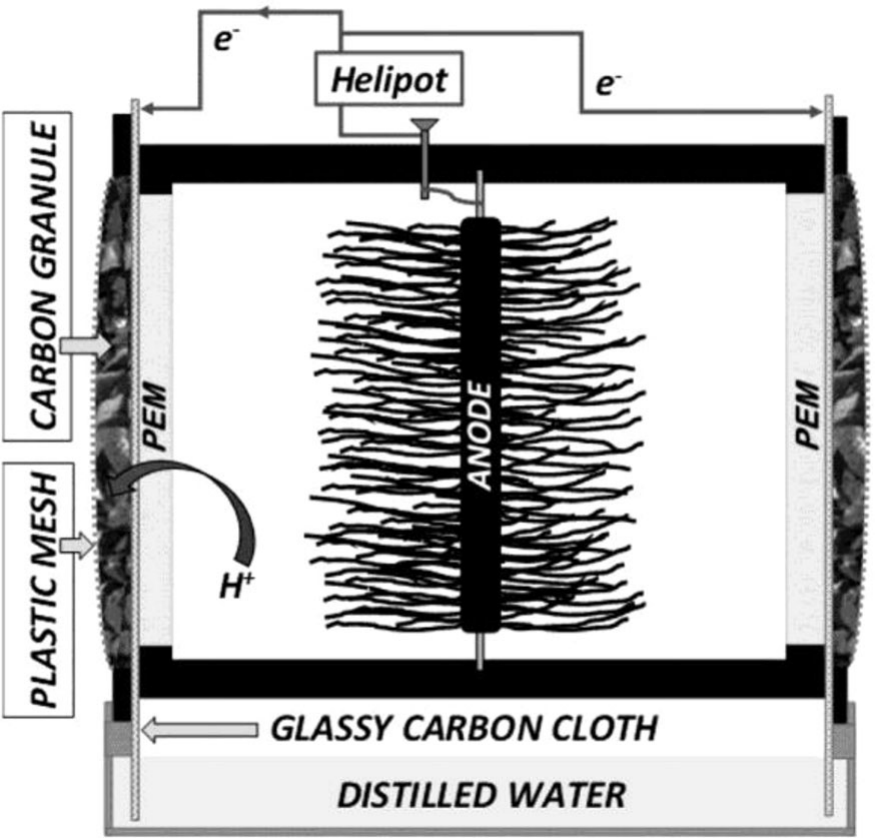
Scheme of the applied MFCs

The parts of the three single chamber air–cathode MFCs (see Fig. 1) applied in this study were developed and provided by the Okinawa Institute of Science and Technology Graduate University—Biological Systems Unit, Okinawa, Japan. The MFCs with an inner volume of 230 cm^3^ each were assembled from prefabricated PVC parts.

Graphite brushes with a diameter of 10 cm were used as anode. The proton exchange membranes (PEM) were porous ceramic plates with ion exchange polymer in the pores. The air–cathodes consisted of 3 layers. The first layer was a glassy carbon cloth that was immersed in distilled water during the whole operation time supplying the necessary aqueous medium for the oxygen reduction reaction through capillary action, and also providing the dilution and removal of soluble contaminants (e.g. salts) possibly diffusing from the anode chamber through the ceramic layer. The current collecting wires were connected to this glassy carbon layer through titanium screws. The second layer of the air cathode was made of a new, patented, biomass-based renewable material: 25 ml carbonized coconut shell granules (diameter cca. 2–4 mm) were applied based on Schaffer-Harris et al. 2017. The reduction of the oxygen takes place on the surface of these granules. Finally, a plastic mesh was applied to fix the other 2 layers and membrane surface together, maintaining the connection with the PEM. The geometrical contact surface of the PEM with the air cathode was 48 cm^2^. Each cell had two identical air cathodes on the two opposite sides that were connected in parallel. Two tube connection point for influent and effluent, as well as a sealable sampling hole were placed on the surface of the cell.

The electronic apparatus included the circuit, the adjustable external resistance (helipot, 0–10 kΩ) and data collection device (Graphtec midi logger GL840 oscilloscope). Potential on the external resistor was measured and registered in every 20 s. Electric parameters (current, power) were calculated from voltage values.

Anodic chambers of all three MFCs were connected hydraulically to the same 1 l stirred buffer tank by plastic tubing with an inner diameter of 1.5 mm, thus, the total liquid volume of the system was 1690 ml not counting the negligible volume of the tubing. Circulation of the anolyte between the buffer tank and the anode chambers was provided by a peristaltic pump (Masterflex^®^). MFCs and the buffer tank were kept in a thermostat (WTW 606) to maintain the desired temperature. During the whole research, the three MFCs (referred as Cell “A”, Cell “B” and Cell “C”) were operated under the same conditions in order to investigate the reproducibility of the results in parallel systems, and to be able to calculate basic statistics (e.g. standard deviation).

### MFC operation

#### Composition of the media

During general operation, media in the anodic chamber and the buffer tank contained salt components as follows: 3.13 g l^−1^ NaHCO_3_, 0.31 g l^−1^ NH_4_Cl, 0.13 g l^−1^ KCl, 4.22 g l^−1^ NaH_2_PO_4_, 6.93 g l^−1^ Na_2_HPO_4_ × 12 H_2_O plus trace elements (based on Oh et al. 2004). Sodium-acetate or peptone (Molar chemicals) was added as carbon source. Standard concentration of the sodium acetate was 40 mM (2550 mg COD l^−1^) during inoculation and basic investigation of the cell parameters (polarization measurements). To maintain anaerobic conditions, before introducing to the anode chamber, fresh media was de-oxygenated by heating up to ~ 70 °C and then cooled down to room temperature while bubbling with N_2_ continuously.

#### Inoculation

Primary settled sludge from domestic wastewater treatment plant was used as inoculum. Before introducing to the anode chamber, it was diluted to ~ 1 g TSS l^−1^ (total suspended solids) with the salt solution described previously, containing 40 mM acetate substrate. Anodic chambers and the buffer tank were filled with a total volume of 1690 ml inoculating suspension, flow rate of the media between the buffer tank and each cell was 690 ml h^−1^, resulting in 20 min of hydraulic retention time. Within a ~ 2-weeks long inoculation period applying 1000 Ω external resistance, the voltage increased and stabilized at 0.5–0.6 V in each cell. Having the voltage stabilized, the inoculating suspension was washed out with fresh media.

#### Polarization measurements

Polarization tests were carried out to determine the basic electric parameters of the cells (maximum power output, internal resistance). To eliminate the effect of a possible hysteresis, polarization tests were carried out first by increasing the adjustable external resistance from 0 to 10,000 Ω in specified steps, after that, decreasing the resistance from 10,000 to 0 Ω using the same steps. Polarization curves were obtained by calculating the average values for each external resistance.

#### Determination of concentration dependence of the current

Anode chambers of the MFCs were washed through with 4 l of fresh de-oxygenated carbon source free media in order to reach substrate free conditions. 1 l of the same, substrate free media was introduced to the buffer tank and circulated between the MFCs and the buffer tank. Because of substrate free operation, the potential of the cells decreased below 2–5 mV (depending on the external resistance), which was considered to be the threshold value of endogenous metabolism in this experiment. Having reached the endogenous regime, stepwise increasing of the acetate concentration (thus increasing the available carbon source in the fuel cells) was carried out by the addition of small volumes (typically 1–4 ml) of high concentration (6500 mg COD l^−1^) sodium acetate solution to the stirred buffer tank. To guarantee the appropriate mixing of the increased acetate concentration media of the buffer with the media in the anodic compartments, flow rate between the buffer tank and each cell was adjusted to 1380 ml h^−1^, resulting in 10 min of hydraulic retention time in the anodic compartments during this experiment. Samples for acetate concentration measurements were collected from the anolyte at least 30 min following the acetate addition in order to provide the required time for the concentration equalization between the buffer and anode compartments of the MFCs.

Acetate concentrations from centrifuged (19,000 g, Hermle Z323) samples taken from the anolyte were measured by a Shimadzu gas chromatograph with flame ionization detector, AOC 22i automatic injector and Restek Stabilwax DA 30 m × 0.32 mm ×  0.25 μm capillary column. The initial column temperature was 100 °C, that was increased by 10 °C/min up to 210 °C, then kept at 210 °C for 10 min. Hydrogen was used as carrier gas with a flow rate of 50 ml/sec. Temperature of FID was 280 °C. 3-pointed calibration was used for quantitative determination.

## Results and discussion

### Performance characteristics of the MFCs at 30 °C temperature

**Fig. 2:**
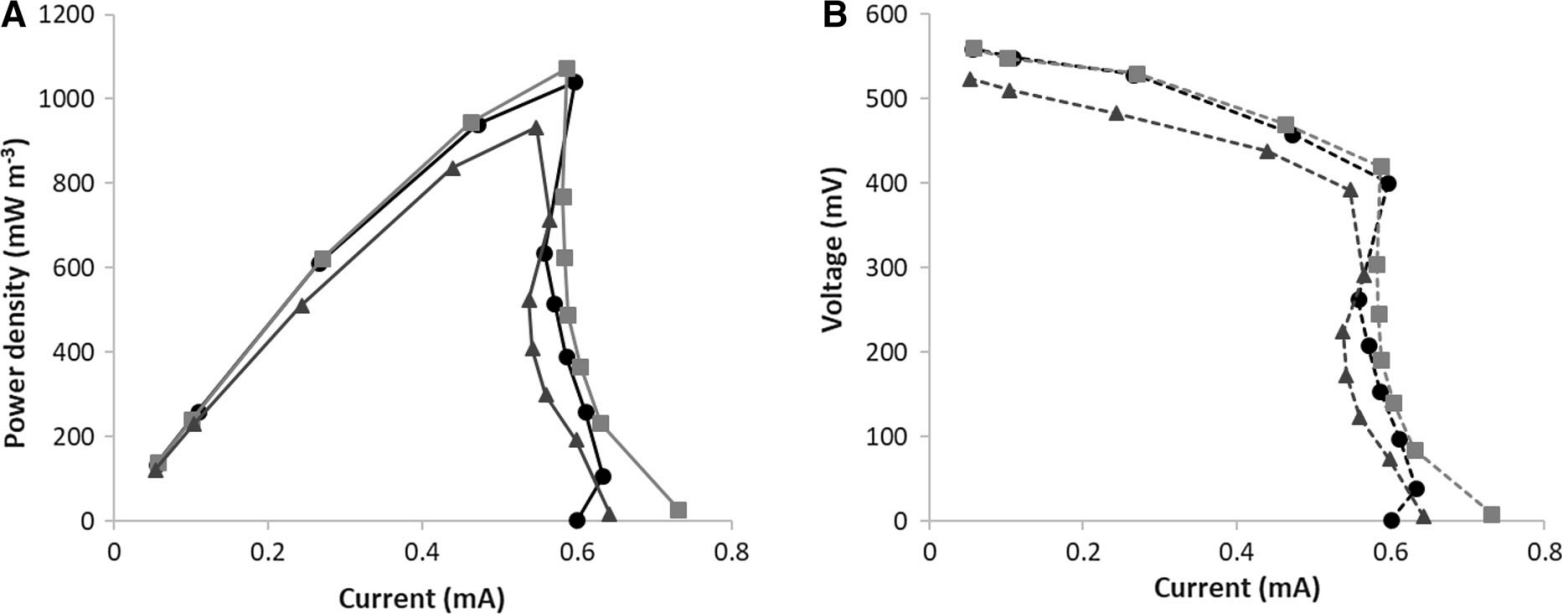
Power density (**a**); Voltage (**b**) as a function of current in the three MFCs at 30 °C. **a**, **b** : Cell “A”;  : Cell “B”;  : Cell “C”

Typical polarization curves measured in the cells are depicted in Fig. 2, at 30 °C. Power density values were calculated by normalizing the power obtained in the cells to the anolyte volume (2.3 × 10^−5^ m^3^). It can be observed that the three cells produced very similar polarization characteristics. Shape of both the power density and the voltage curves, however, is slightly different compared to the results obtained in two-chambered MFCs in our previous studies (Tardy et al. 2017a, b), as a remarkable drop can be observed both in the power density and voltage at ~ 0.6 mA current (~ 600 Ω external resistance). This phenomenon was observed in several previous studies (e.g. Min et al. 2008; Ieropoulos et al. 2010). The suggested explanation is mass transport limitations (e.g. proton transport in the PEM), or the so-called “power overshoot” theory: the depletion of ions and electrons in the anolyte as a result of the low external resistance (Ieropoulos et al. 2010).

**Fig. 3:**
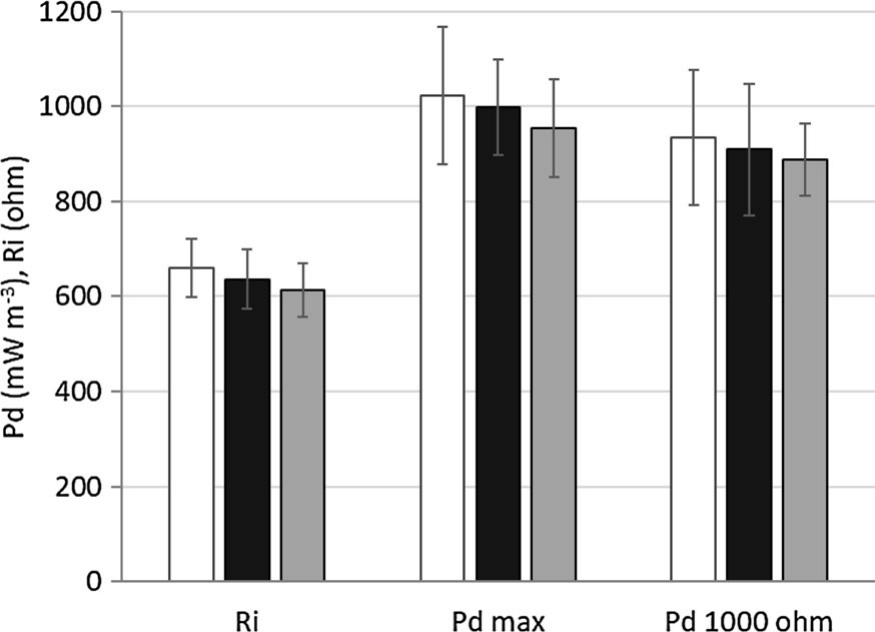
Internal resistance (R_i_), maximum power density (P_d max_) and power density measured at 1000 Ω external resistance (P_d 1000Ω_) values obtained in the MFCs.  : MFC “A”;  : MFC “B”;  : MFC “C”

Basic performance parameters of the cells (internal resistance; maximum power density; power density at 1000 Ω external resistance) are depicted in Fig. 3. These values were calculated from four polarization measurements carried out on four different days. Between the polarization measurements the MFCs were operated by applying R_e_ = 1000 Ω (R_e_—external resistance).

Calculated average internal resistance values of the three cells were in the range of 610–660 Ω, the standard deviation in each cell remained below 10% of the average value. Maximum power density averages of the cells (measured by applying the value of internal resistance as external resistance: R_e_ = R_i_) were in the range of 958–1023 mW m^−3^, while power density averages at R_e_ = 1000 Ω were in the range of 887–933 mW m^−3^. These values are in accordance with the values obtained for MFCs with similar design (Logan et al. 2015; Rahimnejad et al. 2015). No significant difference was observed (α = 0.05) between the investigated cells for all of the three parameters, so the studied MFCs showed stable and similar operational characteristics. The investigated parameters were continuously monitored over the total ~ 5 months of experimental period and no considerable shift was observed.

### Temperature dependence of current generation

**Fig. 4:**
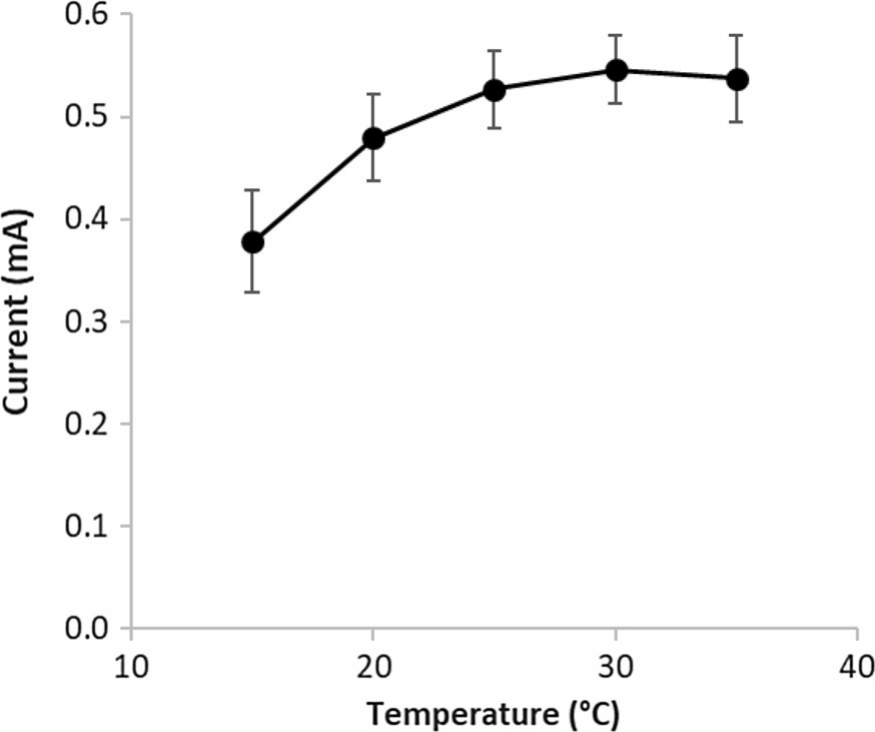
Current generated in the cells (external resistance: 1000 Ω) as a function of operating temperature

In order to verify the effect of the temperature on the biodegradation rate, and as a result, on the generated current (see Eq. 1), the performance of the cells was investigated on 5 different temperatures (15; 20; 25; 30 and 35 °C, respectively) applying 1000 Ω external resistance (see Fig. 4). Highest current (0.55 ± 0.03 mA) was observed at 30 °C, however, with the calculated standard deviation, current values are not significantly different in the 25 to 35 °C temperature range (α = 0.05). At lower temperatures, the current generation drops, especially at 15 °C (to 0.38 ± 0.05 mA). It can be concluded that the temperature tolerance of the biomass in the MFC is mesophilic type, which is not surprising as the inoculation of the cells were carried out with basically mesophilic culture.

### Concentration dependence of the current

**Fig. 5:**
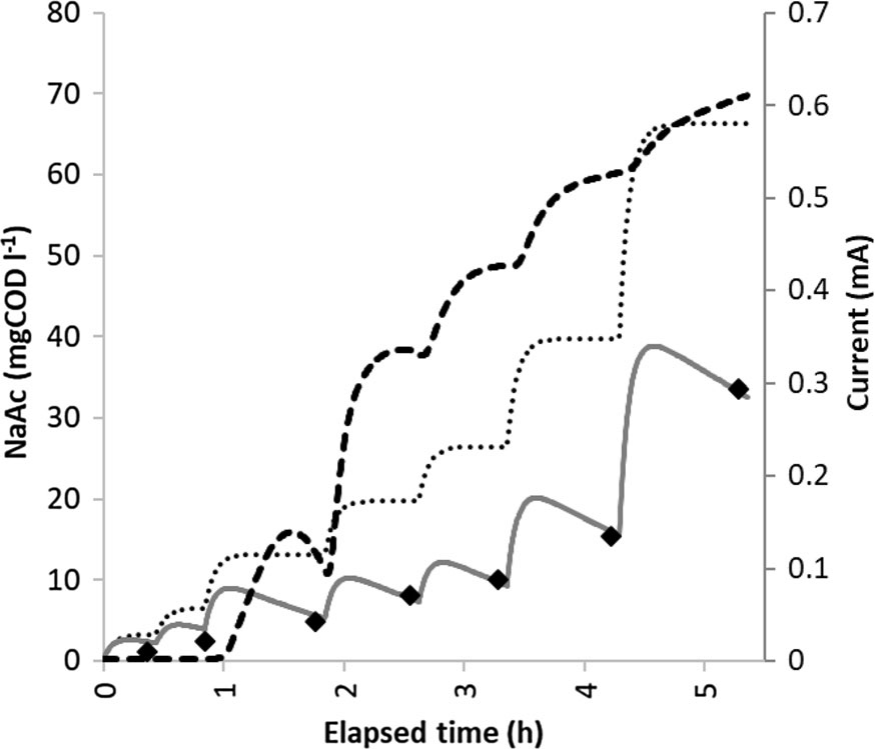
Measured and modeled acetate concentrations and the obtained current as a function of time during the measurement of concentration dependence with 600 Ω external resistance.  : Acetate concentration measured with gas chromatography;  Acetate concentration calculated with the “hydraulic model”;  Acetate concentration calculated with the “biodegradation model”;  Measured current in the external circuit of the MFC

Measurement of acetate concentration dependence of the current with stepwise substrate concentration increase was carried out with three different external resistance values (R_e_ = 100 Ω; 600 Ω; 1000 Ω). Figure 5 shows the measured concentration values with a typical current vs. time curve in MFC “A” (with R_e_ = 600 Ω).

In order to predict the concentration of the acetate continuously during the experiment, assuming no biodegradation, a numeric hydraulic model was developed to use as a reference to measure the impact of biodegradation against. In this hydraulic model, we assumed that the concentration of the acetate in the anodic chambers is affected only by the hydraulics and dilution (the amount of acetate introduced through the influent and washed out with the effluent), the biodegradation during the experiment was neglected.

By calculating the concentration value in every 20 s of the experiment, the hydraulic model showed the expected concentration steps (see Fig. 5, dotted line). Comparing the calculated concentration curve with the measured data, it is obvious that biodegradation has a strong effect on the actual acetate concentration in the anolyte. To be able to describe the concentration appropriately, the hydraulic model was complemented with the calculation of biodegradation of acetate with a Monod-kinetics based model. The acetate quantity removed by biodegradation over the 20 s time frame can be calculated by Eq. 2.

2 $$S_{rem} = r_{max} \frac{{S_{MFC} }}{{K_{s} + S_{MFC} }}\Delta t$$

where S_rem_ is the removed acetate amount (mg COD), S_MFC_ is the actual acetate concentration in the anode chamber (mg COD l^−1^), r_max_ is the maximum acetate removal rate (mg COD h^−1^) of the MFCs, K_s_ is the half saturation constant (in the specific case depicted on Fig. 5 it is 6.7 mg COD l^−1^, see Table 1) and ∆t = 20 s equals to 5.55 × 10^−3^ h. r_max_ was measured in the cells at high (> 2000 mg COD l^−1^) substrate concentrations, resulting in 3.6 mg COD h^−1^ value per cell. Biodegradation was implemented in the preceding hydraulic model. Comparing the measured acetate concentrations with this “biodegradation model” results (see Fig. 5, grey line), it can be concluded that the elaborated model appropriately describes the acetate concentration in time (average difference between measured and modeled values is less than 2 mg COD l^−1^).

**Table 1:** Parameters of the Monod-type concentration dependence model of the current

R_e_ (Ω)	I_max_ (mA)	K_S_ (mg COD l^−1^)
100	1.29	8.2
600	0.96	6.7
1000	0.57	4.3

R_e_: External resistance; I_max_: maximum current; K_S_: half saturation constant

Comparing the measured current values (see Fig. 5 dashed line) with the modeled acetate concentration values, it can be concluded that after the substrate deficient state, the exoelectrogenic biomass does not produce considerable current at extremely low acetate concentrations (< 5 mg COD l^−1^). Exceeding acetate concentration of ~ 5 mg COD l^−1^ (third acetate dosage step), the current profile follows the stepwise concentration changes.

**Fig. 6:**
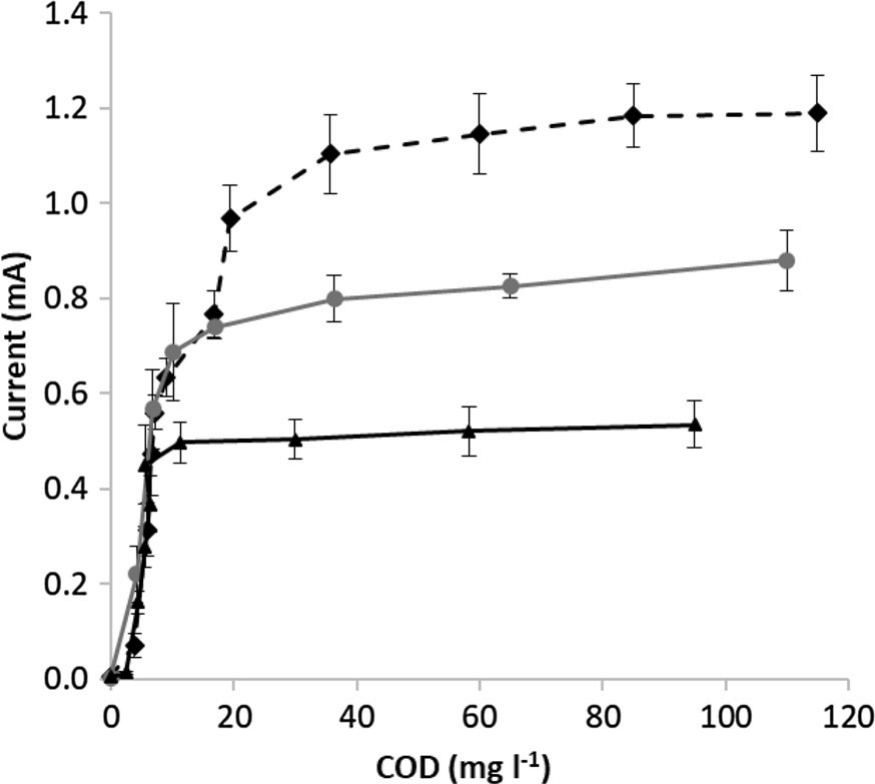
Obtained average current of the operated 3 MFCs with the standard deviation as a function of acetate concentration at three different external resistance (Re) values.  : 100 Ω external resistance;  : 600 Ω external resistance;  : 1000 Ω external resistance

Depicting the obtained current values as a function of measured acetate concentrations at three different external resistance values (100, 600 and 1000 Ω, respectively, see Fig. 6) supports the assumption that acetate concentration dependence of the current follows Monod-like kinetics (see Eq. 1). Monod curves were fitted to the measurement results, the obtained maximum current and half saturation constant values are summarized in Table 1. Current depends on the external resistance, so I_max_ values increase with the decreasing R_e_ values. Although, by decreasing the external resistance K_S_ values slightly increased, even in case of the lowest 100 Ω external resistance, the half saturation constant remained below 10 mg COD l^−1^. With 100 Ω of R_e_, baseline current of the substrate free state is 20 ± 3 µA (originating from the endogenous metabolism). Measurements showed that acceptable signal strength for detection (higher than 30 µA) occurred typically at ≥ 5 mg COD l^−1^ concentration, thus this value can be considered as detection limit of this measurement. As a result of the low K_S_ value, considerable concentration dependence of the current can be observed only in the range of low acetate concentrations (< 40 mg COD l^−1^, see Fig. 6), where the sensitivity of the cell provides higher than 5 µA current change per mg COD l^−1^ substrate concentration change. At higher substrate concentrations, the sensitivity decreases and above 70 mg COD l^−1^ current becomes practically independent from the acetate concentration (current obtained at 70 mg COD l^−1^ acetate concentration is higher than 90% of the I_max_ value).

**Fig. 7:**
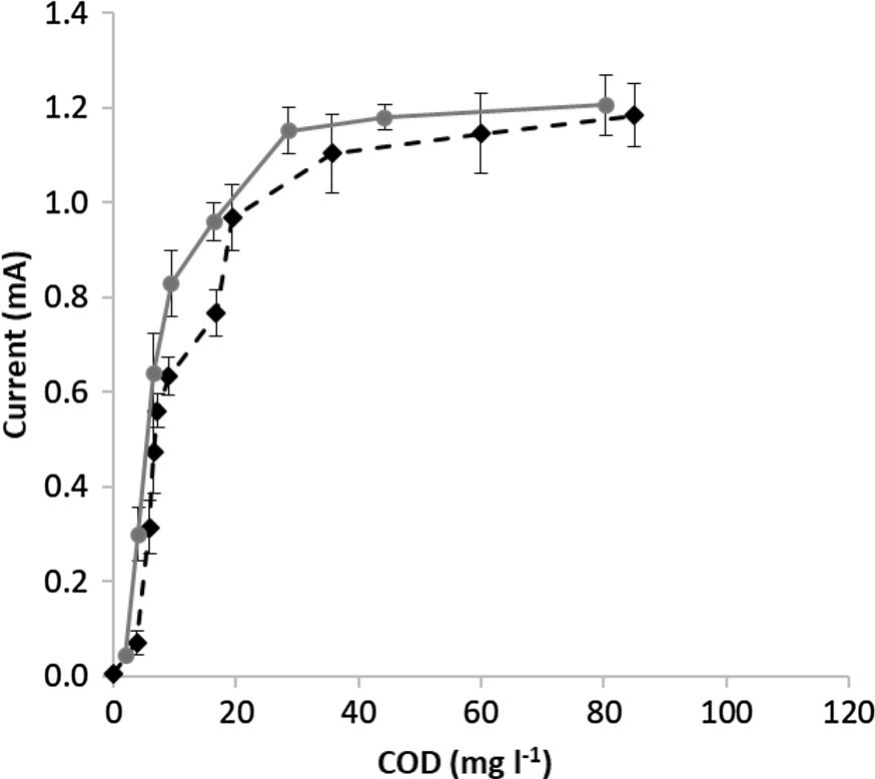
Obtained average current of the operated 3 MFCs with the standard deviation as a function of COD concentration of the media with acetate and peptone as carbon source.  : Acetate based media;  : Peptone based media

In order to investigate the concentration dependence with a complex substrate at 100 Ω external resistance, the measurement was repeated by using peptone (Molar Chemicals) as carbon source instead of acetate. Comparing the measured current values (see Fig. 7), it can be concluded that the shape of the curves is similar for the synthetic (acetate based) and the complex (peptone based) media. Kinetic parameter values of 6.8 mg COD l^−1^ K_S_ and 1.33 mA I_max_ calculated by fitting Monod-curves to the measured current values obtained for peptone based media confirm the suggestion that biodegradation of the complex peptone-based media occurring with practically the same kinetics as the synthetic acetate.

## Conclusions

Single chamber air cathode microbial fuel cells were investigated for application as biosensors for the determination of biodegradable organics concentration. The MFCs with the applied biomass originated cathode material and with the developed design operated with a steady and well-reproducible performance at high (> 2000 mg COD l^−1^) acetate concentrations, providing an average of 1014 mW m^−3^ maximum power density and higher than 0.5 V potential and 0.5 mA current, guaranteeing appropriate signal strength for biosensor application at 1000 Ω external resistance.

Inoculating with mesophilic biomass originated from pre-clarified sludge of a domestic wastewater treatment plant, stable current was observed in the range of 25–35 °C temperature range. Below this range at 15 °C, however, the biodegradation rate (and as a result, the current) in the MFCs decreased significantly. Based on this result, it can be suggested, that if biosensors with this MFC design are operated in non-thermostated environment, the effect of the temperature has to be taken into consideration (e.g. temperature dependent calibration has to be carried out) for the appropriate operation, especially below 20 °C.

Acetate concentration dependence of the current obtained in the MFCs showed the expected Monod-type relationship. Decreasing the external resistance from 1000 Ω to 100 Ω caused an increase (from 0.57 to 1.29 mA) in the I_max_ values. Half saturation constant, however, remained below 10 mg COD l^−1^ with all the investigated external resistance values. Concentration dependence of the current showed similar characteristics by applying peptone as complex carbon source instead of acetate.

Results suggest that biosensors with the investigated MFC design and operation are potentially applicable for detecting as low as 5 mg COD l^−1^ readily biodegradable substrates, and the concentration of these substances can be calculated directly from current up to ~ 50–70 mg COD l^−1^ in natural waters or wastewater treatment plant effluents.
